# Circulating tumor cells: a valuable marker of poor prognosis for advanced nasopharyngeal carcinoma

**DOI:** 10.1186/s10020-019-0112-3

**Published:** 2019-11-15

**Authors:** Guoping Ou, Shan Xing, Jianpei Li, Lin Zhang, Shulin Chen

**Affiliations:** Department of Laboratory Medicine, State Key Laboratory of Oncology in South China, Collaborative Innovation Center for Cancer Medicine, Guangdong Key Laboratory of Nasopharyngeal Carcinoma Diagnosis and Therapy, Sun Yat-sen University Cancer Center, Guangzhou, 510060 People’s Republic of China

**Keywords:** Nasopharyngeal carcinoma, Circulating tumor cells, Epstein-Barr virus DNA, Prognosis, Cut-off point

## Abstract

**Purpose:**

To evaluate the prognostic value of circulating tumor cells (CTCs) in nasopharyngeal carcinoma (NPC).

**Methods:**

Cox’s proportional hazards regression models were used to identify whether CTCs was a poor prognostic factor for NPC. Chi-square tests were used to analyze and compare the distribution characteristics of CTCs in NPC. ROC curve was used to estimate the cut-off point of CTCs. Kaplan-Meier survival analyses were used to observe the prognostic value of CTCs alone and in combined with Epstein-Barr Virus DNA (EBV-DNA).

**Results:**

CTCs was confirmed to be an independent risk factor for poor prognosis of NPC by Cox’s regression models that enrolled 370 NPC cases and took age, gender, EBV-DNA and CTCs as variables. The proportion of CTCs in stage IV NPC was statistically different from that in stage III; the cut-off point of CTCs between stage IV (288 cases) and stage III (70 cases) NPC estimated by ROC curve was 0.5. The prognosis of advanced NPC patients became worse with the increase of CTCs count. The combined detection of CTCs and EBV-DNA could better predict the prognosis of NPC compared with the single detection of EBV-DNA.

## Introduction

Circulating tumor cells (CTCs) count using the CellSearch system (Janssen Diagnostics, Raritan, NJ, USA) has been proved to have good prognostic value for metastatic breast cancer (Bidard et al. 2014). Due to expensive cost of CTCs examination and regional characteristics of nasopharyngeal carcinoma (NPC), the application of CTCs in NPC is still in exploratory stage (Si et al. 2016; Zhang et al. 2018; He et al. 2017); Meanwhile, the detection methods of CTCs were also different in different studies. Compared with Epstein-Barr Virus DNA (EBV-DNA), the application value of CTCs in NPC is controversial (He et al. 2017; Vo et al. 2016). The reasons for different conclusions may be that the detection methods of CTCs or the research approaches adopted by different studies were different, or there were racial differences among patients enrolled in different studies. In this study, paired data of CTCs and EBV-DNA were analyzed by Kaplan-Meier survival analyses to confirm the role of CTCs in the prognosis of NPC. This study focused on whether CTCs has prognostic value for NPC and how to use CTCs to predict the prognosis of NPC patients. It should be noted that the patients enrolled in this study were mainly from southern China.

## Instrument and reagent

**Instrument:** CellTracks® AutoPrep® system and CellTracks® Analyzer II.

**Manufacturer:** Janssen Diagnostics, LLC.

**Kit name:** CellSearch Circulating Tumor Cell Kit (Epithelial).

**Detection principle:** The detection kit is based on ferromagnetic fluid capture reagent and immunofluorescence reagent; the ferromagnetic fluid reagent contains nanoparticles with magnetic cores, the polymer layer around the magnetic core is coated with antibodies against EpCAM antigens to capture CTCs. After the CTCs has been captured and enriched by the immune magnet, fluorescent reagents are added to identify and count CTCs. The fluorescence reagent contains the following components: anti-CK-phycoerythrin (PE) specific to cytokeratin (characteristic of epithelial cells), DAPI for nuclear staining and anti-CD45-phycocyanin (APC) specific to leukocyte.

CellTracks® Analyzer II automatically scan the entire surface of the sample box, capture image and present any fluorescent glow event by CK-PE together with DAPI to the user. If its morphological characteristics are consistent with those of tumor cells, and the immune typing is shown as EpCAM+, CK+, DAPI+, and CD45-, it will be classified as tumor cells.

**Limitations of the method:** If the patient is receiving doxorubicin, the blood should not be drawn until at least 7 days after doxorubicin injection. If a blood sample is taken within 7 days of doxorubicin administration, the interpretation of CellSearch® test results must be cautious. CellSearch® can’t detect CTCs without expression of EpCAM or cytokeratin-8, 18 or 19.

**Interpretation of test results:** The reported test result was <CTCs count>/7.5 ml of blood.

## Subjects information

The cases data enrolled in this study were from primary or recurrent tumor patients who visited our hospital from August 2015 to March 2018. All patients who underwent CTCs examination during this period were analyzed in this retrospective study, including patients with NPC or other tumor types. Clinicians mainly considered the patients’ clinical stages when applying for CTCs examination, and were more inclined to perform CTCs assessment on patients with advanced cancer. The moment of CTCs assessment during the treatment varies, from 2981 days after to 39 days before the first treatment, details of the assessment moment were shown in Additional file 1: Figure S1. Treatments before or after CTCs assessment mainly included chemotherapy and radiotherapy; Some patients with distant metastases would undergo local surgery for the site of metastases, such as radiofrequency ablation, vertebroplasty and lymph node dissection; A few patients participated in clinical trials of new drugs such as triprizumab. Since NPC patients were mainly treated with chemotherapy and radiotherapy, and the proportion of patients receiving surgical treatment was relatively low, there was no statistical analysis on surgical treatment in Additional file 1: Figure S1.

For NPC, there were 307 males, aged 15–80 yr, the median age was 45; and 66 females, aged 15–71 yr, the median age was 43. The proportions of NPC patients in each clinical stage were showed in Additional file 2: Table S1.

The copy of EBV-DNA was detected by Cobas Z 480 fluorescence quantitative PCR instrument (The reference value was < 10^3^ copies/ml). Sample collection, processing and detection of CTCs and EBV-DNA were carried out according to the instructions provided by the manufacturer. The staging of NPC and other tumor types analyzed in this study was referred to AJCC tumor staging manual, 7th edition.

## Methods

Overall survival was defined as time from baseline CTCs assessment to death from any cause. Patients without documented evidence of death were censored at the date of last follow-up. Chi-square tests were used to analyze the proportions of CTCs in different clinical stages, age groups and genders. ROC curve analysis was used to estimate the cut-off point of CTCs between stage III and stage IV NPC. Cox’s regression models combined with Kaplan-Meier survival analyses were used to confirm the relationship between elevated CTCs count and poor prognosis of NPC.

## Results

### CTCs is an independent risk factor for poor prognosis of NPC

Cox’s regression models were used to verify whether CTCs was a risk factor for poor prognosis of NPC. CTCs was analyzed together with age, gender and EBV-DNA in this subparagraph. The variables assignment and available cases of the Cox’s regression models were showed in Table 1. Except for 3 cases did not available for Cox’s regression analyses due to incomplete follow-up data, the Cox’s models in this subparagraph analyzed all NPC cases collected by this study, including 288 patients in stage IV and 82 patients in other stages; 55 cases were not enrolled in the Cox’s models which took EBV-DNA as one of the variables due to the absence of EBV-DNA test results. The median survival time of the 71 dead cases was 263 days. CTCs was confirmed to be an independent risk factor affecting the prognosis of NPC by Cox’s regression models in this subparagraph (Table 1).

**Table 1:** Variables assignment and analysis results of Cox’s regression models for NPC

Analysis type	Factor	Grouping and assignment	Available cases		*P*-value	HR	95.0% CI for HRs	
			Death	Censored			Lower	Upper
Univariate	Age	Continuous variable	71	299	.002	1.03	1.01	1.05
	Gender	1 = “male”; 2 = “female”			.017	.36	.16	.83
	Clinical stage	1 = “I”; 2 = “II”; 3 = “III”; 4 = “IV”	71	295	.001	3.14	1.56	6.35
	EBV-DNA	Continuous variable	64	251	.001	1.00	1.00	1.00
	CTCs	Continuous variable	71	299	.000	1.00	1.00	1.00
Multivariate	Age	Continuous variable	64	248	.003	1.03	1.01	1.06
	Gender	1 = “male”; 2 = “female”			.118	.51	.22	1.19
	Clinical stage	1 = “I”; 2 = “II”; 3 = “III”; 4 = “IV”			.002	3.14	1.54	6.41
	EBV-DNA	Continuous variable			.058	1.00	1.00	1.00
	CTCs	Continuous variable			.001	1.00	1.00	1.00

### The distribution characteristics of CTCs in NPC

**Fig. 1:**
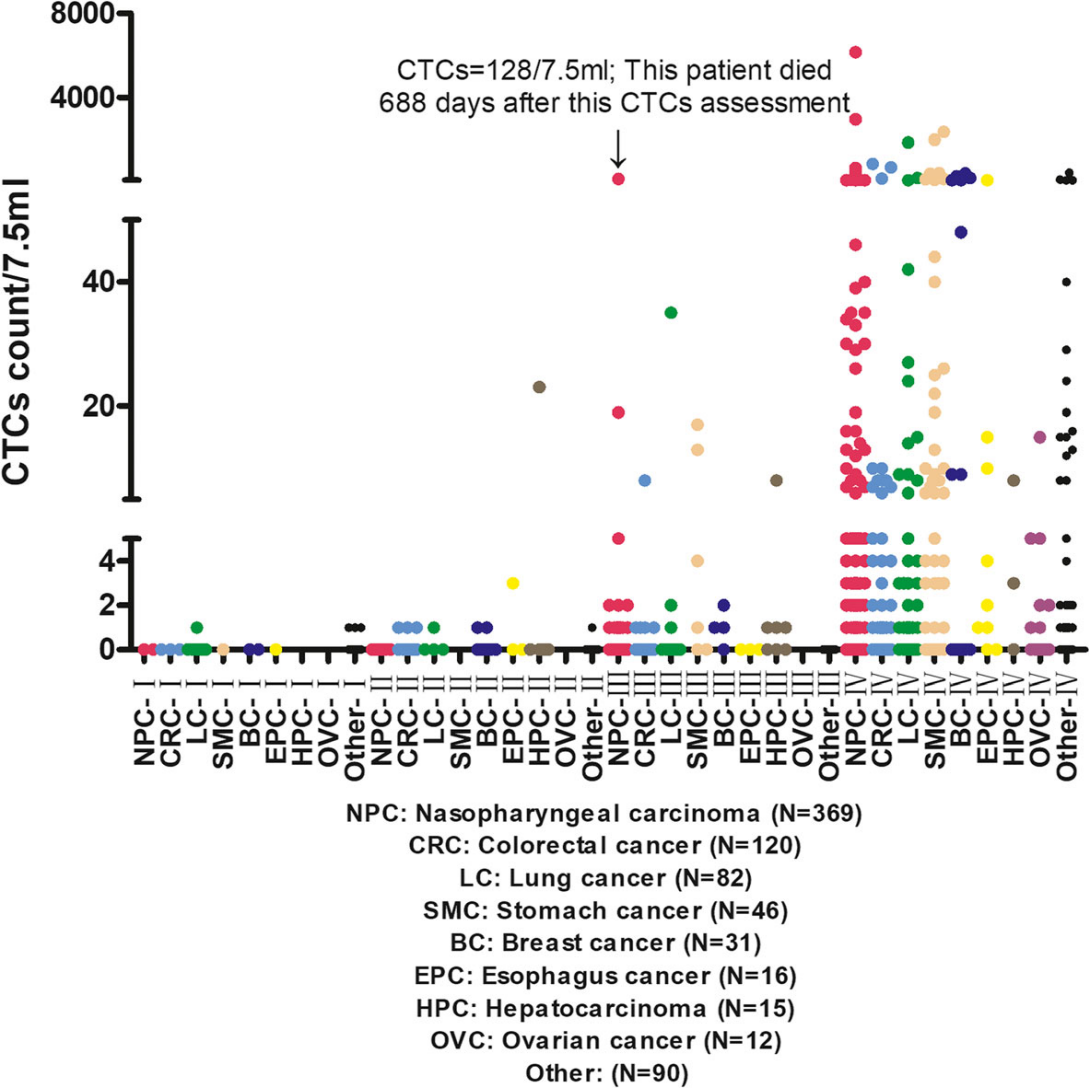
Distribution of CTCs in different tumor types and clinical stages

As a marker of metastasis, CTCs should only exist in advanced tumors theoretically. In practical applications, the CTCs counts of stage III NPC patients were not always 0; In this study, the CTCs counts ranged 1 ~ 128 (70 patients) and 0 ~ 6134 (288 patients) in stage III NPC and stage IV NPC, respectively (Fig. 1). Notably, the patient in stage III and with CTCs = 128 was a male and aged 50y, he died 688 days later. According to case review, when this patient got the test results of CTCs = 128, his EBV-DNA was 1.2*10^4^ copies/ml. He was suspected to have hepatic metastasis of NPC or hepatic hemangioma by color ultrasound, while MR scan was more likely to consider inflammatory lesions. From the final outcome of this patient, perhaps clinicians should pay more attention to CTCs count, CTCs may appear in the bloodstream early in tumor metastasis.

Each tumor type has its own biological characteristics, so the shedding characteristic of CTCs may also be different. Some type of tumor cells may be more easily circulating in the blood (Nguyen et al. 2009). To better observe the distribution characteristics of CTCs in NPC, the proportions of CTCs in NPC were counted, and were compared with those in other tumor types (Fig. 1 and Table 2). As can be seen from Fig. 1, the CTCs counts of most patients in stage III were within 2, while the CTCs count range of patients in stage IV was significantly expanded. Chi-square test showed that, the proportion of CTCs in stage IV NPC was statistically different from that in stage III (Table 2).

**Table 2:** Proportions of CTCs in each NPC clinical stage and their comparison

Stage	I	II	III	IV
CTCs count				
= 0	2	9	54	169
= 1	0	0	10	42
= 2	0	0	3	13
= 3	0	0	0	12
> =4	0	0	3	52
Number of cases				
Total number of cases	2	9	70	288
*P*-value of Chi-square Test:	–		0.011	

**Fig. 2:**
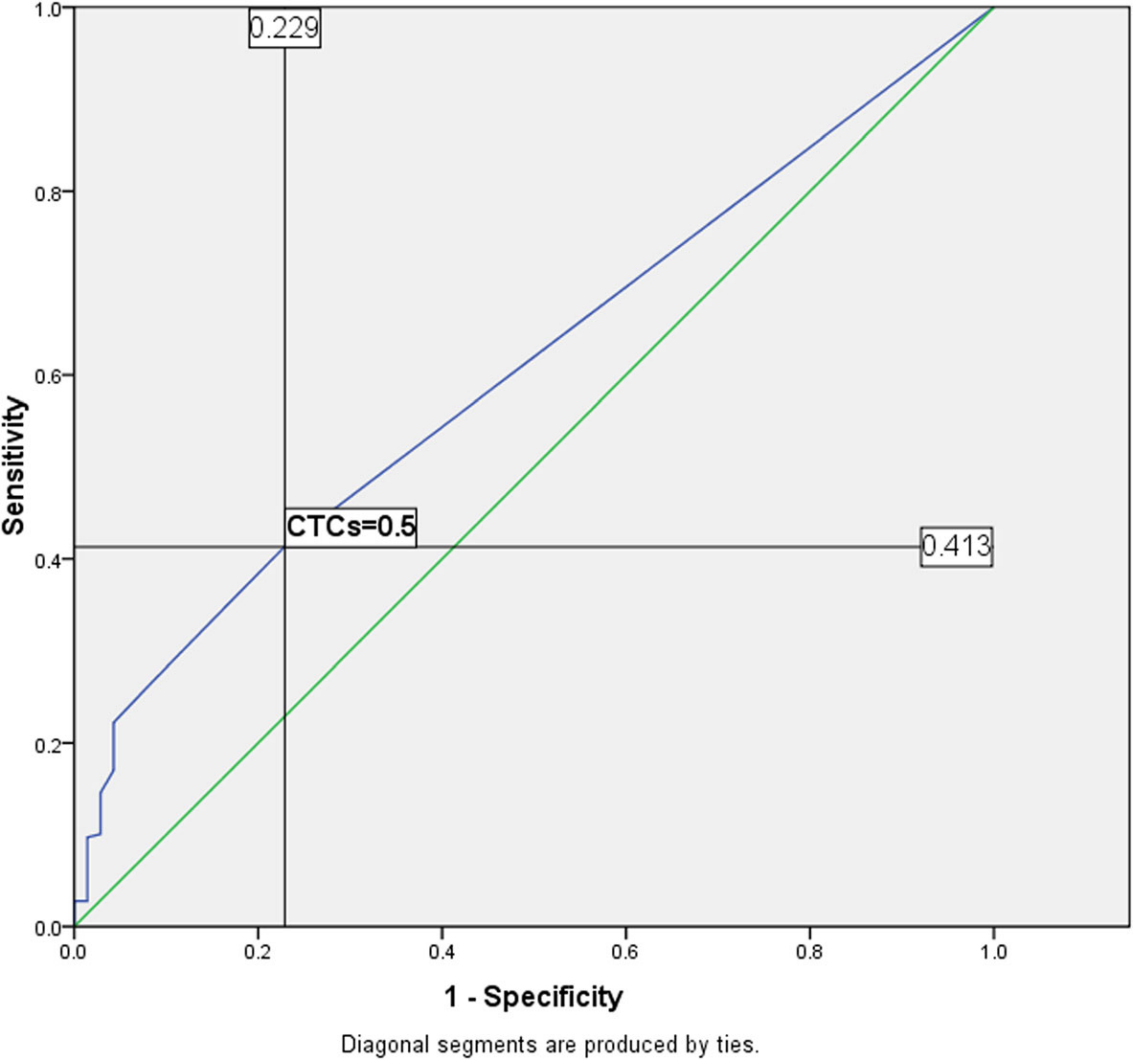
ROC curve of NPC-(III and IV) based on CTCs

In order to observe the difference of CTCs counts between stage III and stage IV NPC, and give clinicians a reference to judge the patients’ condition, ROC curve was used to analyze the CTCs counts of NPC in stage III and stage IV (70 cases and 288 cases, respectively, Fig. 2). The result showed that the cut-off point of CTCs between stage III and stage IV NPC was 0.5, and the area under the ROC curve was 0.608. Details of the ROC curve were shown in Additional file 3: Table S2, indicating that the statistics about the ROC curve in this subparagraph may be biased.

### Prognostic value observation of CTCs

In view of the factors that affecting the prognosis of NPC also include age and gender (Xiao et al. 2013; Proceedings of the 7th Biannual International Symposium on Nasopharyngeal Carcinoma 2015 n.d.), the proportions of CTCs in different age groups and genders were also analyzed here (Additional file 4: Table S3 and Additional file 5: Table S4). As can be seen from Additional file 4: Table S3, although the prognosis of male NPC patients was worse than that of female (OuYang et al. 2015), there was no statistical difference in the proportions of CTCs between male and female. From Additional file 5: Table S4, although the difference was not significant, it seemed that tumor cells of elderly advanced NPC patients were more likely to be shed from the primary site into the bloodstream, resulting in increased CTCs count; As opposed to that, there was no statistical difference in the proportions of CTCs between the two age groups (<45y and > =45y) in stage III NPC, suggesting that CTCs did not increase with age in stage III NPC.

**Fig. 3:**
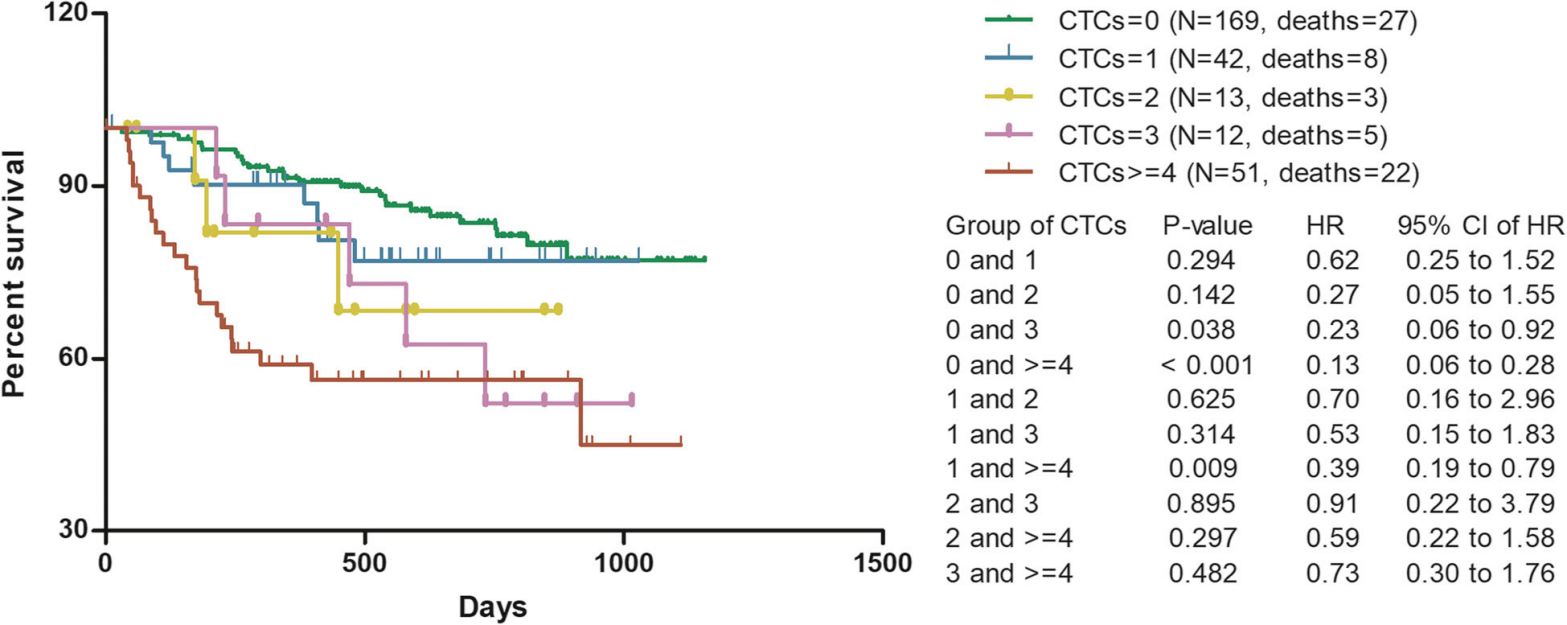
Survival of advanced NPC grouped by CTCs count

Kaplan-Meier analyses were performed for 287 stage IV NPC patients (288 cases in total, 1 case lacked follow-up data). It showed that the prognosis of advanced NPC patients became worse with the increase of CTCs count (Fig. 3). As shown in Fig. 3, perhaps because the current cases number cannot assess the optimal cut-off point, there was no statistical difference between CTCs = 0, CTCs = 1 and CTCs = 2 in predicting the prognosis of NPC. To avoid the interference of clinical stage on the observation of prognostic value and exploration of cut-off point, Kaplan-Meier survival analyses in this subparagraph were only performed for advanced NPC patients.

### Combined detection of CTCs and EBV-DNA can better predict the prognosis of NPC

The role of EBV-DNA in the prognosis of NPC has been confirmed (Liu et al. 2017; Prayongrat et al. 2017). The cut-off point of EBV-DNA vary among different studies, such as 8000 copies/ml or 2300 copies/ml (Chai et al. 2012; Lertbutsayanukul et al. 2018). The patients enrolled in the Kaplan-Meier survival analyses in this subparagraph were grouped by EBV-DNA = 4000 copies/ml (Chen et al. 2016) and CTCs = 1/7.5 ml. To avoid the interference of selection bias, Kaplan-Meier analyses in this subparagraph were only performed on paired EBV-DNA and CTCs data. Paired data of EBV-DNA and CTCs was obtained from blood samples taken from the same patient within 3 days, mostly from the same blood sampling.

**Fig. 4:**
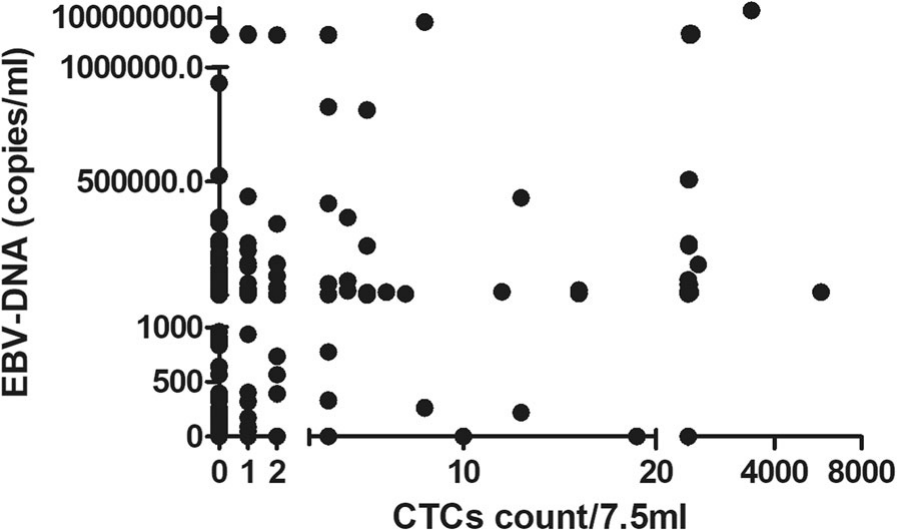
Scatterplot of EBV-DNA associated with CTCs

The scatterplot showed that CTCs and EBV-DNA did not have a good positive correlation (Fig. 4). When CTCs = 0, the EBV-DNA ranged 0 ~ 8.2*10^6^ copies/ml, and the median was 354 copies/ml; When CTCs = 1, the EBV-DNA ranged 0 ~ 7.6*10^6^ copies/ml, and the median was 935 copies/ml; When CTCs = 2, the EBV-DNA ranged 0 ~ 2.22*10^6^ copies/ml, and the median was 1480 copies/ml; When CTCs> 2, the EBV-DNA ranged 0 ~ 1.4*10^8^ copies/ml, and the median was 4.84*10^4^ copies/ml. This poor positive correlation suggested that the two indicators were preferred to appear at different stages of the NPC course, although both CTCs and EBV-DNA were associated with poor prognosis of NPC.

**Fig. 5:**
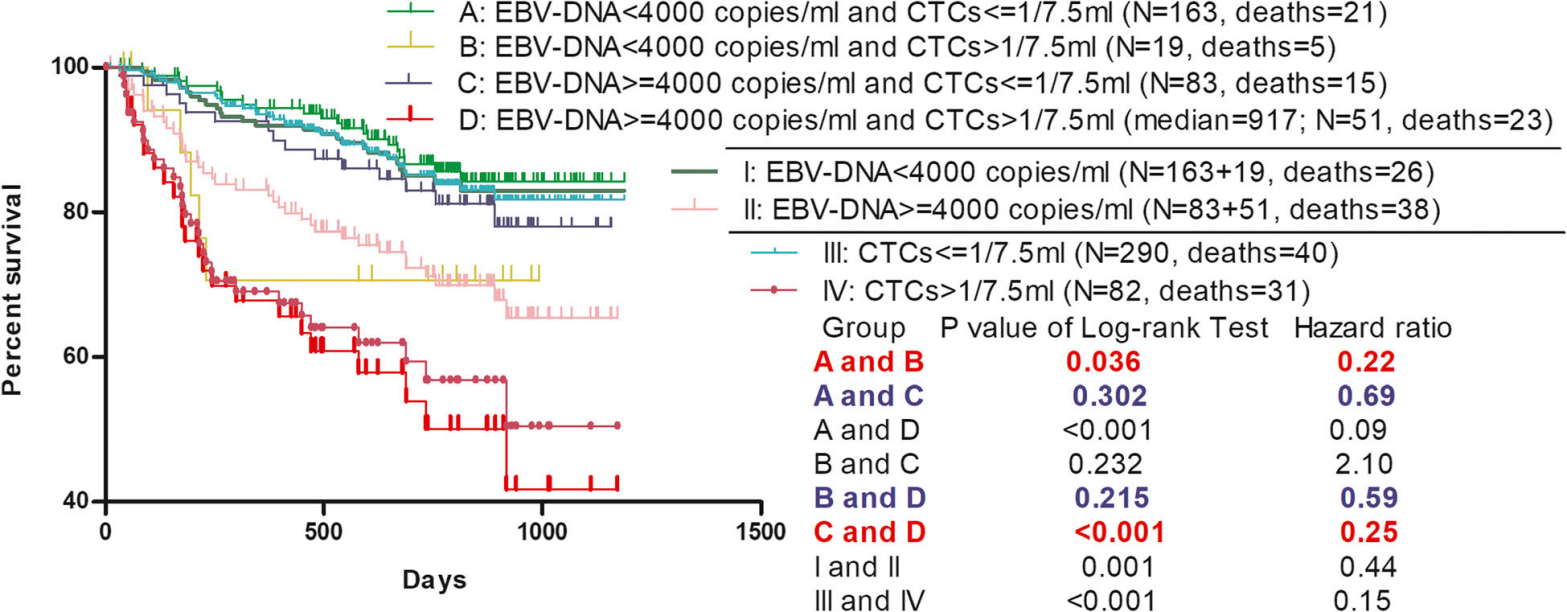
Survival of NPC grouped by EBV-DNA and CTCs = 1/7.5 ml

As could be seen from group I and group II in Fig. 5, NPC patients with EBV-DNA > =4000 copies/ml had significantly worse prognosis than those with EBV-DNA < 4000 copies/ml, this was a predictable result. After CTCs count was added to group those patients above (analyzed in group I and group II), the survival analyses (Group: A, B, C, D) showed that the addition of CTCs as a predictor could better predict the prognosis of NPC. Especially when patients got the test result of EBV-DNA > =4000 copies/ml (Group: II), CTCs was particularly valuable (Group: C, D). Although the cases number of group B was limited, the prognostic value of CTCs in patients with EBV-DNA < 4000 copies/ml (Group: I) could still be observed (Group: A, B). It could be seen from Fig. 5 that the combined detection of CTCs and EBV-DNA could better predict the prognosis of NPC compared with the single detection of EBV-DNA.

## Discussion

A study from Singapore showed that there was no statistically significant relationship between CTCs and clinical stages (Vo et al. 2016). However, data analyses in this study showed that there was a statistical difference between the proportions of CTCs in stage III and stage IV NPC (Table 2). The study from Singapore used a technology named Microsieve to detect CTCs (Lim et al. 2012). The Microsieve technology was a size-based method capable of isolating both epithelial and mesenchymal CTCs, utilizing the size and deformability differences between the CTCs and normal blood cells; In contrast, Cellsearch system captured CTCs by nanoparticles with magnetic cores which were coated with antibodies against EpCAM antigens. The fundamental difference between Microsieve and Cellsearch was the way CTCs were captured, that was, size-based or EpCAM-based. Besides, the definition of CTCs was not quite the same between Microsieve and Cellsearch; Microsieve classified CK+, CD45- nucleated cells as canonical CTCs, while Cellsearch classified EpCAM+, CK+, DAPI+, and CD45- nucleated cells as tumor cells. In our opinion, the most important reason for the two studies to reach different conclusions was the different ways of capturing CTCs.

Although Cox’s regression models in subparagraph 1 and Kaplan-Meier survival analyses in subparagraph 4 analyzed all available cases collected by this study, regardless of gender and clinical stage, most of the cases analyzed in these two subparagraphs were male patients with stage IV NPC. Therefore, the conclusions of this study are best applicable to male advanced NPC.

In the multivariate Cox’s regression model, only 64 patients (20.5%) were followed up to the end event (Death), this may affect the accuracy of statistical results. Since the survival time of NPC patients is generally long, this problem is inevitable now. This exploratory study suggests that CTCs is a possible prognostic biomarker. But its definitive evaluation, above all, its comparison to EBV-DNA, should be necessarily evaluated in a prospective controlled study, with more focused eligibility criteria.

## Conclusions

Cox’s regression models confirmed that CTCs was an independent risk factor affecting the prognosis of NPC. By analyzing the distribution characteristics of CTCs in NPC and comparing the differences of different CTCs counts in predicting the prognosis of advanced NPC patients, it was confirmed that the prognosis of advanced NPC patients became worse with the increase of CTCs count. Kaplan-Meier survival analyses confirmed that the combined detection of CTCs and EBV-DNA could better predict the prognosis of NPC.

## Supplementary information

**Additional file 1: Figure S1.** Assessment moment of CTCs during the treatment of NPC patients.

**Additional file 2: Table S1.** Proportions of patients in each NPC clinical stage.

**Additional file 3: Table S2.** Area under the ROC curve .

**Additional file 4: Table S3.** Proportions of CTCs in different genders of NPC .

**Additional file 5: Table S4.** Proportions of CTCs in different age groups of NPC .

## Data Availability

The datasets analyzed during the current study are not publicly available due to patient privacy concerns, but are available from the corresponding author on reasonable request.

## Abbreviations

CTCs: Circulating tumor cells
EBV-DNA: Epstein-Barr Virus DNA
NPC: Nasopharyngeal carcinoma

## References

[CR1] Bidard FC, Peeters DJ, Fehm T (2014). Clinical validity of circulating tumour cells in patients with metastatic breast cancer: a pooled analysis of individual patient data. Lancet Oncol.

[CR2] Chai SJ, Pua KC, Saleh A (2012). Clinical significance of plasma Epstein-Barr virus DNA loads in a large cohort of Malaysian patients with nasopharyngeal carcinoma. J Clin Virol.

[CR3] Chen WH, Tang LQ, Guo SS (2016). Prognostic value of plasma Epstein-Barr virus DNA for local and regionally advanced nasopharyngeal carcinoma treated with Cisplatin-based concurrent Chemoradiotherapy in intensity-modulated radiotherapy era. Medicine (Baltimore).

[CR4] He C, Huang X, Su X (2017). The association between circulating tumor cells and Epstein-Barr virus activation in patients with nasopharyngeal carcinoma. Cancer Biol Ther.

[CR5] Lertbutsayanukul C, Kannarunimit D, Netsawang B (2018). Optimal plasma pretreatment EBV DNA cut-off point for nasopharyngeal cancer patients treated with intensity modulated radiation therapy. Jpn J Clin Oncol.

[CR6] Lim LS, Hu M, Huang MC (2012). Microsieve lab-chip device for rapid enumeration and fluorescence in situ hybridization of circulating tumor cells. Lab Chip.

[CR7] Liu TB, Zheng ZH, Pan J, Pan LL, Chen LH (2017). Prognostic role of plasma Epstein-Barr virus DNA load for nasopharyngeal carcinoma: a meta-analysis. Clin Invest Med.

[CR8] Nguyen DX, Bos PD, Massagué J (2009). Metastasis: from dissemination to organ-specific colonization. Nat Rev Cancer.

[CR9] OuYang P, Zhang L, Lan X (2015). The significant survival advantage of female sex in nasopharyngeal carcinoma: a propensity-matched analysis. Brit J Cancer.

[CR10] Prayongrat A, Chakkabat C, Kannarunimit D, Hansasuta P, Lertbutsayanukul C (2017). Prevalence and significance of plasma Epstein-Barr virus DNA level in nasopharyngeal carcinoma. J Radiat Res.

[CR11] Yogyakarta, Indonesia. Proceedings of the 7th Biannual International Symposium on Nasopharyngeal Carcinoma 2015. BMC Proceedings. 2016;10(Suppl 1).

[CR12] Si Y, Lan G, Deng Z (2016). Distribution and clinical significance of circulating tumor cells in nasopharyngeal carcinoma. Jpn J Clin Oncol.

[CR13] Vo JH, Nei WL, Hu M (2016). Comparison of circulating tumour cells and circulating cell-free Epstein-Barr virus DNA in patients with nasopharyngeal carcinoma undergoing radiotherapy. Sci Rep.

[CR14] Xiao G, Cao Y, Qiu X, Wang W, Wang Y (2013). Influence of gender and age on the survival of patients with nasopharyngeal carcinoma. BMC Cancer.

[CR15] Zhang J, Shi H, Jiang T (2018). Circulating tumor cells with karyotyping as a novel biomarker for diagnosis and treatment of nasopharyngeal carcinoma. BMC Cancer.

