# Supplementary figures and images for: Circulating tumor cells: a valuable marker of poor prognosis for advanced nasopharyngeal carcinoma
Source: Mol Med. 2019 Nov 15;25:50. doi: 10.1186/s10020-019-0112-3 (PMC6858770; doi:10.1186/s10020-019-0112-3)

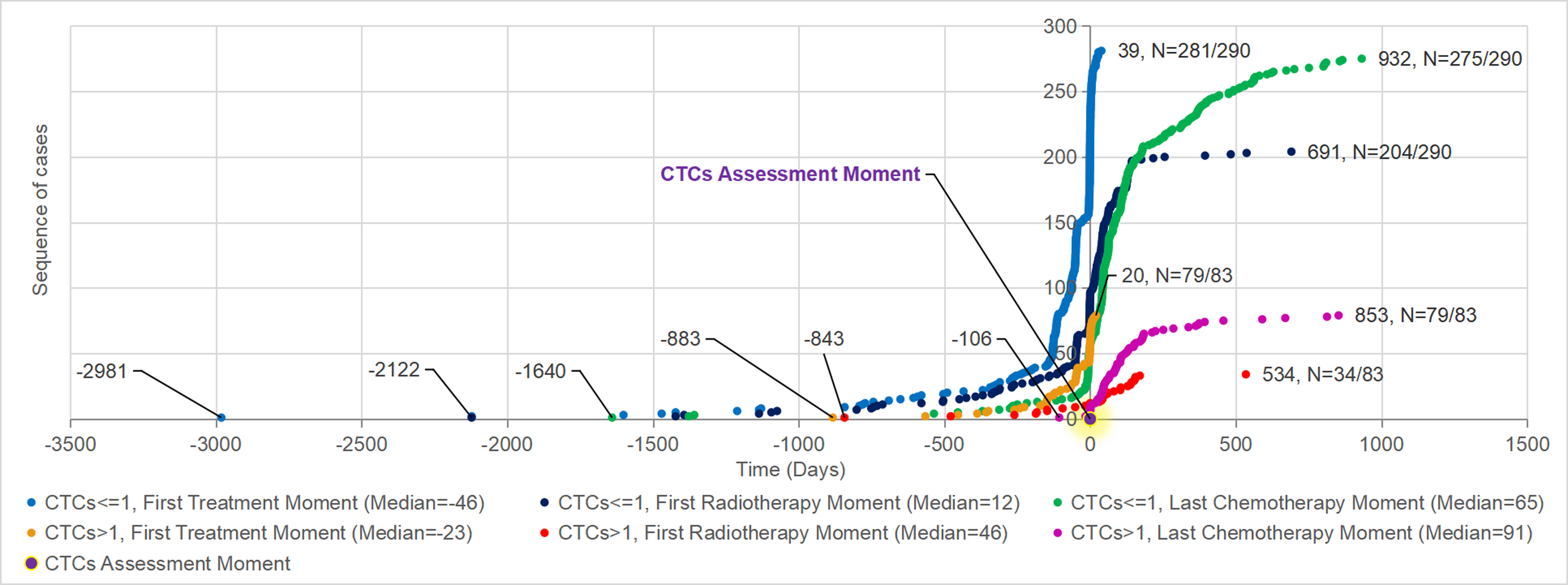

Supplement: Supplementary file 1 — Additional file 1: Figure S1. Assessment moment of CTCs during the treatment of NPC patients. [file 10020_2019_112_MOESM1_ESM.tif]
