# Supplementary material for: Circulating tumor cells: a valuable marker of poor prognosis for advanced nasopharyngeal carcinoma
Source: Mol Med. 2019 Nov 15;25:50. doi: 10.1186/s10020-019-0112-3 (PMC6858770; doi:10.1186/s10020-019-0112-3)
Supplement: Supplementary file 2 — Additional file 2: Table S1. Proportions of patients in each NPC clinical stage. [file 10020_2019_112_MOESM2_ESM.docx]

| **Table S1. Proportions of patients in each NPC clinical stage** | | | | | | |
| --- | --- | --- | --- | --- | --- | --- |
| Stage | I | II | III | IV | unclear | P-value of Chi-square Test grouped by gender |
| Male number | 1 | 5 | 52 | 245 | 4 | 0.023 |
| Female number | 1 | 4 | 18 | 43 | 0 |  |
