# Supplementary material for: Circulating tumor cells: a valuable marker of poor prognosis for advanced nasopharyngeal carcinoma
Source: Mol Med. 2019 Nov 15;25:50. doi: 10.1186/s10020-019-0112-3 (PMC6858770; doi:10.1186/s10020-019-0112-3)
Supplement: Supplementary file 3 — Additional file 3: Table S2. Area under the ROC curve . [file 10020_2019_112_MOESM3_ESM.docx]

| **Table S2. Area under the ROC curve** | | | | |
| --- | --- | --- | --- | --- |
| Area | Std. Error^a^ | Asymptotic P-value^b^ | Asymptotic 95% CI | |
|  |  |  | Lower Bound | Upper Bound |
| .608 | .034 | .005 | .541 | .675 |
| The test result variable(s): CTCs has at least one tie between the positive actual state group and the negative actual state group. Statistics may be biased.  a. Under the nonparametric assumption  b. Null hypothesis: true area = 0.5 | | | | |
