# Supplementary material for: Circulating tumor cells: a valuable marker of poor prognosis for advanced nasopharyngeal carcinoma
Source: Mol Med. 2019 Nov 15;25:50. doi: 10.1186/s10020-019-0112-3 (PMC6858770; doi:10.1186/s10020-019-0112-3)
Supplement: Supplementary file 4 — Additional file 4: Table S3. Proportions of CTCs in different genders of NPC . [file 10020_2019_112_MOESM4_ESM.docx]

| **Table S3. Proportions of CTCs in different genders of NPC** | | | | | | | | | |
| --- | --- | --- | --- | --- | --- | --- | --- | --- | --- |
| Stage | | I | | II | | III | | IV | |
| Gender | | male | female | male | female | male | female | male | female |
| CTCs count | =0 | 1 | 1 | 5 | 4 | 39 | 15 | 145 | 24 |
|  | =1 | 0 | 0 | 0 | 0 | 7 | 3 | 34 | 8 |
|  | =2 | 0 | 0 | 0 | 0 | 3 | 0 | 12 | 1 |
|  | =3 | 0 | 0 | 0 | 0 | 0 | 0 | 11 | 1 |
|  | >=4 | 0 | 0 | 0 | 0 | 3 | 0 | 43 | 9 |
| Number of cases Total number of cases | | 1 | 1 | 5 | 4 | 52 | 18 | 245 | 43 |
| P-value of Chi-square Test: | | － | | － | | 0.514 | | 0.771 | |
