# Supplementary material for: Circulating tumor cells: a valuable marker of poor prognosis for advanced nasopharyngeal carcinoma
Source: Mol Med. 2019 Nov 15;25:50. doi: 10.1186/s10020-019-0112-3 (PMC6858770; doi:10.1186/s10020-019-0112-3)
Supplement: Supplementary file 5 — Additional file 5: Table S4. Proportions of CTCs in different age groups of NPC . [file 10020_2019_112_MOESM5_ESM.docx]

| **Table S4. Proportions of CTCs in different age groups of NPC** | | | | | | | | | |
| --- | --- | --- | --- | --- | --- | --- | --- | --- | --- |
| Stage | | I | | II | | III | | IV | |
| Age group | | <45y | >=45y | <45y | >=45y | <45y | >=45y | <45y | >=45y |
| CTCs count | =0 | 1 | 1 | 3 | 6 | 29 | 25 | 92 | 77 |
|  | =1 | 0 | 0 | 0 | 0 | 3 | 7 | 25 | 17 |
|  | =2 | 0 | 0 | 0 | 0 | 2 | 1 | 4 | 9 |
|  | =3 | 0 | 0 | 0 | 0 | 0 | 0 | 5 | 7 |
|  | >=4 | 0 | 0 | 0 | 0 | 0 | 3 | 19 | 33 |
| Number of cases Total number of cases | | 1 | 1 | 3 | 6 | 34 | 36 | 145 | 143 |
| P-value of Chi-square Test: | | － | | － | | 0.159 | | 0.065 | |
